# Bacterial community associated with the surface and inside of centipede forcipules: Identification and characterization

**DOI:** 10.1371/journal.pone.0341165

**Published:** 2026-01-16

**Authors:** Yasutaka Tanaka, Daiki Mizushima, Yoshimitsu Izawa, Tomohiro Matsumura, Chikara Yonekawa, Hirotomo Kato, Takashi Mato

**Affiliations:** 1 Department of Emergency and Critical Care Medicine, Jichi Medical University, Tochigi, Japan; 2 Department of Infection and Immunity, Division of Medical Zoology, Jichi Medical University, Tochigi, Japan; Makerere University College of Natural Sciences, UGANDA

## Abstract

**Background:**

In tropical to subtropical regions, centipede bites may prompt medical attention, with manifestations largely reflecting venom-related discomfort, although infections, including rare fatal necrotizing soft tissue infection (NSTI), have been reported. However, no reports are available on the commensal bacteria on centipede forcipules.

**Objectives:**

This study aimed to investigate bacterial species residing on and in centipede forcipules and their potential role in post-bite infections.

**Methods:**

Nine *Scolopendra mutilans*, three *Scolopendra japonica*, and two *Bothropolys rugosus* were collected from three regions in Japan. The bacterial composition of their forcipules was analyzed using 16S ribosomal ribonucleic acid gene sequencing and microbiome analysis.

**Findings:**

A diverse bacterial community was observed on the centipede forcipules. Among the NSTI-associated genera examined (*Escherichia, Staphylococcus,* and *Streptococcus*), only *Staphylococcus* was identified as a minor population.

**Conclusion:**

This study provides the first evidence that some bacteria found on centipede forcipules have been previously isolated from centipede bite infections. The risk of infection from bacteria on centipede forcipules during a centipede bite appears low. However, the presence of diverse bacterial species emphasizes the importance of thoroughly cleaning centipede bite wounds to prevent secondary infection.

## Introduction

Over 3,500 described species of centipedes occur globally [1]. Centipedes are widely distributed from the tropics to the subtropics, and they prefer dark, damp environments [1]. Centipedes bite animals using venom claws known as forcipules, which are modified front legs rather than fangs in animals such as snakes. In tropical to subtropical regions, centipede bites are a common reason for seeking medical attention. Most symptoms from centipede bites are primarily attributed to their venom, which can cause swelling, pain, lymphadenitis, cardiac arrhythmia, and acute renal failure [2,3]. However, severe necrotizing soft tissue infections (NSTIs), including fatal cases, have been reported following centipede bite [4–7]. Although such infections are rare, identifying the causative bacteria and administering appropriate antimicrobial treatment are crucial.

Previous studies have reported Methicillin-Resistant *Staphylococcus Aureus* and Group A *Streptococcus* as causative bacteria of NSTI following centipede bites [4–7]. However, the origin of these bacteria remains unclear; it is uncertain whether the infections are caused by human skin commensals or resident bacteria from the centipede. To date, no reports are available on commensal bacteria on centipede forcipules. Substances with antibacterial activity are present in centipede body extracts and venom [8–10]; therefore, specific bacteria may be selectively associated with centipede forcipules. If bacteria from centipede forcipules invade the bite site and cause an infection, identifying the resident bacteria on the forcipules becomes essential. This study characterized the bacterial community on centipede forcipules to provide valuable insights into the treatment of bacterial infections following centipede bites.

## Materials and methods

### Sample collection

The Institutional Animal Experiment Committee of Jichi Medical University formally granted an exemption from ethical review. In this study, nine *Scolopendra mutilans* L. Koch (*Sm*), three *Scolopendra japonica* L. Koch (*Sj*), and two *Bothropolys rugosus* L. Koch (*Br*) underwent bacterial analysis of the forcipules. The centipedes were captured between January and April 2022 from three locations in Japan: a riverside wood area in Sakado City, Saitama Prefecture (two *Sj* and five *Sm*); a wood area in Shimotsuke City, Tochigi Prefecture, located approximately 90 km from Sakado City (two *Sm* and two *Br*); and a wood area in Ako City, Hyogo Prefecture, located approximately 410 km from Sakado City (one *Sj* and two *Sm*). The centipedes were immobilized by cold anesthesia on ice in sterile Petri dishes and were euthanized by decapitation. Following euthanasia, we removed the forcipules of the centipedes with sterilized scissors under a microscope. The forcipules were placed in 100 μL of a saline solution in a microtube and spun in a vortex mixer for 1 min to extract surface-associated microbes. We dissected a central section from one Sj specimen and a small amount of intestinal fluid was dissolved in 100 µL of a saline solution. Moreover, we collected a small amount of soil from a centipede collection site in Tochigi Prefecture and dissolved it in 100 µL of a saline solution.

The collection site was not located within a protected area such as a national park, and the centipedes investigated are not classified as protected arthropods. Therefore, no specific sampling permits were required.

### Incubation on a blood agar plate and identification by 16S rRNA gene sequence analysis

The microbial sample (50 μL) containing resident bacteria present on the surface of the forcipules and in the venom glands was spread on a blood agar plate (Kohjin Bio Co., Saitama, Japan) and incubated at 37°C for up to 5 days; the temperature was chosen to reflect human body conditions and to assess potential pathogenic relevance to humans. The resulting colonies were subjected to species identification using 16S ribosomal ribonucleic acid (rRNA) gene sequence analysis. The colonies obtained after incubation were subjected to polymerase chain reaction (PCR) to identify the bacteria. Colonies were collected using a micropipette tip and dissolved in 10 μL of saline in a microtube. Subsequently, 0.5 μL of the solution was used for PCR amplification. Amplification of the bacterial 16S rRNA-encoding gene was conducted using primers 10F (GTTTGATCCTGGCTCA) and 800R (TACCAGGGTATCTAATCC) as well as AmpliTaq Gold deoxyribonucleic acid (DNA) polymerase (Thermo Fisher Scientific K.K., Tokyo, Japan). A total of 30 PCR cycles were performed, with each cycle comprising melting at 95°C for 60 s, annealing at 55°C for 60 s, and extension for 60 s at 72°C. Before the first cycle, the samples were heated at 95°C for 5 min, and the final cycle was extended to 10 min at 72°C. We confirmed that the DNA had been amplified by electrophoresing 1 μL of each PCR product on an agarose gel.

The PCR products were purified using a FastGene Gel/PCR Extraction Kit (NIPPON Genetics, Tokyo, Japan) to remove excess primers. The purified products were labeled with the 10F primer using the BigDye Terminator v.3.1 Cycle Sequencing Kit (Applied Biosystems, Foster City, CA, USA) and analyzed on the Genetic Analyzer 3500 DNA Sequencer (Thermo Fisher Scientific). The obtained nucleotide sequences were searched using BLAST (https://blast.ncbi.nlm.nih.gov/Blast.cgi) to identify the bacteria.

### Analysis of bacterial flora using comprehensive 16S rRNA gene sequencing

The remaining 50 μL of the microbial sample was stored at −70°C and subjected to microbiome analysis using comprehensive 16S rRNA gene sequencing. The sample was homogenized in a 2.0-mL tube containing 50 μL of the sample (including the venom claws) and 450 μL of a saline solution, which had been stored at −70°C after vortexing. Zirconia beads/φ3mm stainless steel beads were added to the mixture and mechanically crushed using a ShakeMan6 bead crusher (Bio Medical Science, Tokyo, Japan). The crushed cells were centrifuged, and a cell residue precipitate was obtained. DNA was obtained as 300 μL of supernatant and purified using a ReliaPrep DNA Cleanup and Concentration System kit (Promega Corporation, Madison, WI, USA) according to the manufacturer’s instructions. The Illumina MiSeq Sequencing protocol [11] was followed.

We performed two-step PCR to amplify the variable region (V3-V4) of the 16S rRNA-encoding gene using the primers 341F and 806R (Table 1). The total reaction volume for the PCR reactions was 15 µL. The first PCR was performed for 35 cycles, and the second PCR was performed for 20 cycles using AmpligTaq Gold 360 DNA polymerase. The PCR conditions were as follows: initial denaturation at 95°C for 5 min, followed by melting at 95°C for 30 s, annealing at 55°C for 30 s, and extension for 30 s at 72°C in each cycle. The final cycle was extended for 5 min at 72°C. Each PCR product was reamplified using the index primers (Table 2). Amplicon libraries were prepared for Illumina sequencing. The amplified products were electrophoresed on a 1.5% agarose gel and sequenced using the Illumina MiSeq platform and the MiSeq reagent kit version 3 (Illumina, Inc., San Diego, CA, USA).

**Table 1:** 16S rRNA amplification primers.

Primer Name	5’-	Overhang	Illumina adaptor	16S rRNA V3-V4 universal	−3’
16S_V3-V4_341F		TCGTCGGCAGCGTC	AGATGTGTATAAGAGACAG	CCTACGGGNGGCWGCAG	
16S_V3-V4_806R		GTCTCGTGGGCTCGG	AGATGTGTATAAGAGACAG	GGACTACNVGGGTWTCTAAT	

**Table 2:** Index primers.

Primer Name	5’-	Flow cell binding sequences	Index sequences	Overhang	−3’
P7-R001		AATGATACGGCGACCACCGAGATCTACAC	ACCTGCAA	TCGTCGGCAGCGTC	
P7-R002		AATGATACGGCGACCACCGAGATCTACAC	GTTCCTTG	TCGTCGGCAGCGTC	
P7-R003		AATGATACGGCGACCACCGAGATCTACAC	CCAGATCT	TCGTCGGCAGCGTC	
P7-R004		AATGATACGGCGACCACCGAGATCTACAC	AAGTGTGA	TCGTCGGCAGCGTC	
P7-R005		AATGATACGGCGACCACCGAGATCTACAC	CCATGATC	TCGTCGGCAGCGTC	
P7-R006		AATGATACGGCGACCACCGAGATCTACAC	TCATGTCT	TCGTCGGCAGCGTC	
P7-R007		AATGATACGGCGACCACCGAGATCTACAC	TTCGTGGT	TCGTCGGCAGCGTC	
P7-R008		AATGATACGGCGACCACCGAGATCTACAC	GGAACGTA	TCGTCGGCAGCGTC	
P7-R009		AATGATACGGCGACCACCGAGATCTACAC	TGTCAGTC	TCGTCGGCAGCGTC	
P7-R010		AATGATACGGCGACCACCGAGATCTACAC	AAGATCAC	TCGTCGGCAGCGTC	
P7-R011		AATGATACGGCGACCACCGAGATCTACAC	GTTGAACG	TCGTCGGCAGCGTC	
P7-R012		AATGATACGGCGACCACCGAGATCTACAC	TTGGTCAG	TCGTCGGCAGCGTC	
P5-F001		CAAGCAGAAGACGGCATACGAGAT	AACCTCTC	GTCTCGTGGGCTCGG	
P5-F002		CAAGCAGAAGACGGCATACGAGAT	CGATGGTA	GTCTCGTGGGCTCGG	

### Statistical analysis

The output reads from the MiSeq system (2 × 301 bp, paired-end) were obtained in the fastq format and imported into Quantitative Insights into Microbial Ecology 2 (QIIME2) (version 2020.6.0) [12]. Paired-end reads were trimmed and merged using the DADA2 module of the QIIME2 Plugin [13]. The sequences were clustered into amplicon sequence variants (ASVs) using QIIME2. The ASVs were annotated according to the SILVA version 138 database [14] to ensure a sequence similarity threshold of at least 99%. These data were subsequently combined and analyzed in R software version 4.2.2 (R Foundation for Statistical Computing, Vienna, Austria) using the qiime2R, phyloseq, and MicrobiotaProcess packages [15–17].

## Results

### Commensal bacteria associated with the surface and inside of centipede forcipules based on sequence analysis of the 16S rRNA-encoding gene following incubation on a blood agar plate

We analyzed 13 centipede forcipules, excluding one *Sm* from Tochigi Prefecture. Over 10 colonies were observed in seven samples, and ≤3 colonies were observed in three samples. The bacterial species in each sample are presented in Table 3. The dominant bacterial species in each sample are underlined. No identical bacteria were detected between the centipede species (*Sj*, *Sm*, and *Br*).

**Table 3:** Bacteria residing in the centipede forcipules, as determined by the sequence analysis of 16S rRNA genes after incubation on blood agar plate.

Sample name of centipede	Area	Centipede/sample	Top hit in NCBI BLAST (identity %, accession number)	Identification (accession number)
S_Sj_1	Saitama Prefecture	*Sj*	*Staphylococcus succinus*[100%, MF582543.1], *Staphylococcus xylosus*[100%, CP188055.1], *Staphylococcus pseudoxylosus*[100%, PV810820.1], *Staphylococcus saprophyticus*[100%, MW193758.1]	*Staphylococcus* sp. (LC896144)
S_Sj_2	Saitama Prefecture	*Sj*		No valid DNA detected
S_Sm_1	Saitama Prefecture	*Sm*	*Bacillus anthracic*[100%, MH260911.1], *Bacillus cereus*[100%, AY647292.1], *Bacillus tropicus*[100%, MW423424.1]	*Bacillus* sp.(LC896149)
			*Prescottella equi*[100%, PQ782443.1]	*Prescottella* sp.(LC896150)
			*Achromobacter marplatensis*[100%, PV094546.1], *Achromobacter spanius*[100%, MG198685.1], *Achromobacter deleyi*[100%, PV094559.1], *Achromobacter xylosoxidans*[100%, PV428800.1], *Achromobacter insuavis*[100%, PQ036743.1]	*Achromobacter* sp.(LC896151)
S_Sm_2	Saitama Prefecture	*Sm*		No valid DNA detected
S_Sm_3	Saitama Prefecture	*Sm*	*Providencia rettgeri*[100%, MH988778.1], *Providencia vermicola*[100%, PV867013.1], *Provedencia sneebia*[100%, LR738949.1], *Bacillus aerius*[100%, KX834865]	*Providencia* sp./*Bacillus* sp.(LC896163)
			*Ochrobactrum* sp.[100%, CP125969], *Brucella haematophila*[100%, MW454808.1], *Brucella anthropic*[100%, KX774373], *Ochrobactrum soli*[100%, OP204148.1], *Brucella daejeonensis*[100%, ON688689.1], *Brucella intermedia*[100%, ON688679]	*Ochrobactrum* sp./*Brucella* sp.(LC896164)
S_Sm_4	Saitama Prefecture	*Sm*	*Achromobacter spanius*[100%, PP831838.1], *Achromobacter mucicolens*[100%, JX483710.2], *Endophytic bacterium*[100%, KP757683.1], *Alcaligenaceae bacterium*[100%, FJ013341.1], *Betaproteobacteria bacterium*[100%, KT907036.1], *Achromobacter marplatensis*[100%, OQ300045.1], *Achromobacter xylosoxidans*[100%, MK493659.1], *Achromobacter pestifer*[100%, NR_152016.1], *Achromobacter kerstersii*[100%, PP658534.1]	*Achromobacter* sp./*Endophytic* sp./*Betaproteobacteria* sp.(LC896165)
			*Leucobacter chromiireducens*[100%, MN894283.1]	*Leucobacter* sp.(LC896166)
S_Sm_5	Saitama Prefecture	*Sm*	*Pseudomonas aeruginosa*[98.84% JN910248.1]	*Pseudomonas* sp.(LC896167)
S_Sm_1_IF	Saitama Prefecture	Intestinal fluid (S_Sm_1)	*Buttiauxella noackiae*[100%, KT767925.1], *Pantoe* sp.[100%, MH127720.1], *Buttiauxella agrestis*[100%, PV030172.1], *Buttiauxella ferragutiae*[100%, LN824003.1]	*Buttiauxella* sp./*Pantoea* sp. (LC896155)
			*Bacillus anthracic*[100%, KF894697.1], *Bacillus cereus*[100%, OR115613.1], *Bacillus thuringiensis*[100%, OP954775.1], *Bacillus paramycoides*[100%, LC482256.1], *Bacillus subtillis*[100%, MK726359.1], *Bacillus proteolyticus*[100%, OR775572.1]	*Bacillus* sp.(LC896156)
			*Priestia taiwanensis*[99.59%, OL377898.1], *Bacillus vireti*[99.45%, EU834241.1]	*Priestia* sp./*Bacillus* sp.(LC896157)
T_Sm_1	Tochigi Prefecture	*Sm*	*Microbacterium maritypicum*[98.83%, ON819651.1], *Microbacterium oxydans*[98.64% MG890238.1], *Microbacterium algeriense*[98.64%, PV707157.1], *Microbacterium testaceum*[98.64%, KC633948.1]	*Microbactrium* sp.(LC896145)
			*Streptomyces canus*[100%, ON629771.1], *Streptomyces pseudovenezuelae*[100%, MW642149.1]	*Streptomyces* sp.(LC896146)
			*Paenarthrobacter nicotinovorans*[100%, MF796730.1], *Bacillus Safensis*[100%, KC128953.1], *Paenarthrobacter nicotinovorans* [100%, OPP159882.1], *Paenarthrocbacter histidinolovorans*[100%, PQ856804.1], *Micrococcaceae bacterium*[100%, MK308550.1]	*Paenarthrobacter* sp./*Bacillus* sp./*Micrococcaceae* sp.(LC896147)
			*Streptomyces tsukiyonensis*[100%, MK368447.1], *Kitasatospora aureofaciens*[100%, MK073012.1]	*Streptomyces* sp./*Kitasatospora* sp.(LC896148)
T_Br_1	Tochigi Prefecture	*Br*		not cultured
T_Br_2	Tochigi Prefecture	*Br*	*Chitinophaga* sp.[99.57% ON183168.1], *Chitinophaga qingshengii*[98.99% KF150454.1], *Chitinophaga eiseniae*[98.99% LN890157.1]	*Chitinophaga* sp.(LC896158)
			*Prescottella epui*[100%, PQ782443.1], *Rhodococcus* sp.[100%, KX981434.1]	*Prescottella* sp.(LC896159)
T_Soil	Tochigi Prefecture	Soil	*Bacillus luti*[100%, MK373051.1], *Bacillus toyonensis*[100%, OL757835.1], *Bacillus cereus*[100%, KJ016242.1], *Bacillus thuringiensis*[100%, MZ430448.1], *Bacillus wiedmannii*[100%, MT605497.1], *Heyndrickxia acidiproducens*[100%, MN704842.1], *Bacillus proteolyticus*[100%, MN709307.1], *Bacillus paranthracis*[100%, CP169739.1], *Bacillus albus*[100%, OQ911576.1], *Bacillus arachidis*[100%, PQ813870.1], *Bacillus subtillis*[100%, JN411390.1], *Bacillus bombysepticus*[100%, KM081629.1]	*Bacillus* sp.(LC896152)
			*Prescottella equi* [100%, LC020104.1]	*Prescottella* sp.(LC896153)
			*Bacillus* sp[100%, KF040590.1], *Lysinibacillus sphaericus*[100%, KF312283.1]	*Bacillus* sp./*Lysinobacillus* sp.(LC896154)
H_Sj_1	Hyogo Prefecture	*Sj*	*Pseudomonas aeruginosa*[100%, MN096674.1], *Pseudomonas fluorescens*[100%, MT007283.1], *Pseudomonas sihuiensis*[100%, MK007297.1]	*Pseudomonas* sp.*(LC896144)*
H_Sm_1	Hyogo Prefecture	*Sm*	*Delftia tsuruhatensis*[100%, KT229748.1], *Delftia lacustris*[100%, KU726260.1], *Delftia acidovorans*[100%, OZ253549.1]	*Delftia* sp.(LC896168)
H_Sm_2	Hyogo Prefecture	*Sm*	*Stenotrophomonas maltophilia*[100%, KT825727.1], *Stenophomonas pavanii*[100%, MG905278.1], *Stenotrophomonas geniculate*[100%, PV628366.1], *Stenotrophomonas muris*[100%, CP196978], *Gamma proteobacterium*[100%, KT185095.1], *Stenotrophomonas geniculate*[100%, OQ195804.1], *Xanthomonas retroflexus*[100%, KT825693.1]	*Stenotrophomonas* sp./*Gamma* sp./*Xanthomonas* sp.(LC896160)
			*Enterococcus crotali*[100%, MT266930.1], *Enterococcus rotai*[100%, CP157386.1]	*Enterococcus* sp.(LC896161)

The above are results based on bacterial cultures. Some samples wherein DNA extraction was unsuccessful are indicated as “no valid DNA detected.” Some samples wherein no colonies were detected after incubation are indicated as “not cultured.” The dominant bacterial species in each sample are underlined. The dominant bacterial species was defined as the bacterium that formed the most colonies on blood agar. No clear association was observed between bacterial identity and centipede species or capture location.

Centipedes use their forcipules for feeding. *Bacillus* sp*.* was detected dominantly in the intestinal contents of one *Sm* captured in Saitama Prefecture. *Bacillus* sp. was detected in both intestinal and forcipular samples. *Bacillus* sp. was detected dominantly in a soil culture sample from the centipede capture site in Tochigi Prefecture. *Prescottella* sp. and *Bacillus* sp. were detected in both soil and forcipular samples collected in Tochigi Prefecture.

The 16S rRNA sequencing data obtained in this study have been deposited in the DDBJ Nucleotide Sequence Submission System(NSSS) under accession numbers LC896144-LC896168 (Table 3).

### Bacterial flora analysis using comprehensive 16S rRNA gene sequencing

**Fig 1:**
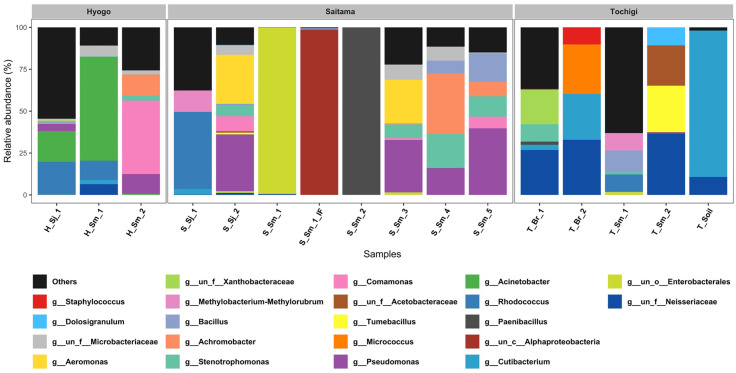
Microbiome analysis of bacterial flora. The 20 most abundant bacterial genus, based on the total read counts across all samples, were plotted for each sample. Less abundant species were grouped into the “Others” category.

We analyzed the microbiota of 14 forcipule, 1 soil, and 1 intestinal fluid samples using comprehensive 16S rRNA gene sequencing and obtained 11,840,742 reads. The top 20 most abundant genera among all reads were plotted for each sampling. Less abundant species were grouped into the “Others” category **(Fig 1**, **Table 4)**. Bacteria comprising over 5% of the total reads in each region were defined as the major constituent bacteria. In six forcipule samples from Saitama (311437 reads), *Enterobacterales* (35.4%), *Pseudomonas* (12.7%), *Rhodococcus* (10.3%), *Achromobacter* (5.6%), and *Stenotrophomonas* (5.3%) were identified as the major component bacteria. In four forcipule samples from Tochigi (385114 reads), *Neisseriaceae* (26.6%), *Cutibacterium* (9.1%), and *Tumebacillus* (8.4%) were detected as the major component bacteria. In three forcipule samples from Hyogo (207132 reads), *Acinetobacter* (29.9%), *Rhodococcus* (11.8%), and *Comamonas* (10.9%) were detected as the major component bacteria.

**Table 4:** Microbiome analysis of bacterial flora. Relative abundances (%) of the top 20 genera across all reads for each sample. Less abundant taxa were grouped into the “Others” category.

Genus	S_Sj_1	S_Sm_4	S_Sm_5	S_Sj_2	H_Sm_2	T_Sm_2	T_Soil	T_Sm_1	S_Sm_1_IF	S_Sm_1	S_Sm_2	T_Br_1	T_Br_2	H_Sm_1	H_Sj_1	S_Sm_3
g__Rhodococcus	45.88	0.00	0.00	0.00	0.00	0.00	0.00	10.43	0.00	0.00	0.00	0.00	0.00	11.50	19.40	0.00
g__un_f__Microbacteriaceae	0.00	8.35	0.48	5.55	2.36	0.00	0.00	0.00	0.00	0.00	0.00	0.00	0.00	6.49	1.12	9.04
g__Micrococcus	0.00	0.00	0.00	0.00	0.00	0.00	0.00	0.00	0.00	0.00	0.00	0.00	29.57	0.00	0.00	0.00
g__Cutibacterium	3.36	0.00	0.00	0.00	0.00	0.00	87.38	0.03	0.05	0.01	0.07	3.13	27.34	2.52	0.12	0.00
g__Tumebacillus	0.00	0.00	0.00	1.05	0.00	27.74	0.00	0.00	0.00	0.00	0.00	0.00	0.00	0.00	0.00	0.00
g__Bacillus	0.28	7.60	16.97	0.82	0.10	0.00	0.00	12.71	0.86	0.00	0.00	0.00	0.00	0.00	0.58	0.24
g__Dolosigranulum	0.00	0.00	0.00	0.41	0.00	10.71	0.00	0.00	0.00	0.00	0.00	0.00	0.00	0.00	0.00	0.00
g__Paenibacillus	0.00	0.00	0.00	0.01	0.00	0.00	0.00	0.00	0.04	0.00	99.93	1.89	0.00	0.00	0.11	0.00
g__Staphylococcus	0.00	0.00	0.00	0.00	0.00	0.01	0.00	0.00	0.00	0.00	0.00	0.39	10.05	0.00	0.00	0.00
g__un_f__Acetobacteraceae	0.00	0.00	0.00	0.86	0.00	24.05	0.00	0.00	0.00	0.00	0.00	0.00	0.00	0.00	0.00	0.00
g__Methylobacterium-Methylorubrum	12.57	0.00	0.00	0.00	0.00	0.00	0.00	10.40	0.00	0.00	0.00	0.00	0.00	0.00	0.00	0.00
g__un_f__Xanthobacteraceae	0.00	0.00	0.00	0.00	0.00	0.00	0.00	0.00	0.00	0.00	0.00	20.70	0.00	0.00	0.36	0.00
g__un_c__Alphaproteobacteria	0.00	0.00	0.00	0.00	0.00	0.00	0.00	0.00	98.52	0.01	0.00	0.00	0.00	0.00	0.00	0.00
g__Aeromonas	0.00	0.00	0.00	29.25	0.00	0.00	0.00	0.00	0.00	0.00	0.00	0.00	0.00	0.00	0.06	26.12
g__Achromobacter	0.00	36.22	8.67	0.03	12.59	0.00	0.00	0.00	0.00	0.00	0.00	0.00	0.00	0.00	0.00	0.34
g__Comamonas	0.00	0.00	6.93	8.91	43.84	0.00	0.00	0.00	0.00	0.00	0.00	0.00	0.00	0.00	0.46	1.16
g__un_f__Neisseriaceae	0.25	0.00	0.00	1.24	0.00	36.57	10.72	0.02	0.00	0.58	0.00	26.79	32.91	6.42	0.05	0.00
g__un_o__Enterobacterales	0.00	0.00	0.00	0.99	0.00	0.00	0.00	1.76	0.00	99.26	0.00	0.00	0.00	0.00	0.06	1.54
g__Acinetobacter	0.00	0.00	0.00	0.05	0.68	0.00	0.00	0.00	0.00	0.00	0.00	0.00	0.00	61.91	18.40	0.00
g__Pseudomonas	0.00	16.07	39.76	33.83	11.77	0.91	0.00	0.00	0.05	0.00	0.00	0.00	0.00	0.00	4.32	31.27
g__Stenotrophomonas	0.00	20.26	12.20	6.56	2.98	0.00	0.00	1.55	0.00	0.00	0.00	10.37	0.00	0.31	0.43	8.10
Others	37.66	11.50	14.99	10.43	25.69	0.00	1.90	63.09	0.48	0.13	0.00	36.73	0.12	10.84	54.53	22.19

We assessed the extent to which the NSTI-associated genera *Streptococcus*, *Staphylococcus*, and *Escherichia* [18] were represented in our results, and only genera_*Staphylococcus* was detected. The bacteria were detected in three of the four centipede samples (T_*Sm*_2, T_*Br*_1, and T_*Br*_2) from Tochigi. However, the proportion was considerably low, at 1.05% of all samples and 0.01% (T_*Sm*_2), 0.39% (T_*Br*_1), and 10.05% (T_*Br*_2) of individual samples.

The intestinal contents of one *S. mutilans* obtained from Saitama Prefecture were dominated by *Alphaproteobacteria* (98% [96,553 reads]). *Cutibacterium* (87%, 67,546 reads), *Neisseriaceae* (10.7%, 8,284 reads), and *Acidibacter* (1.9%, 1,469 reads) were detected in the soil samples from Tochigi Prefecture. *Cutibacterium* and *Neisseriaceae* were also detected in centipede forcipules from the three regions.

The microbiome sequencing data obtained in this study have been deposited in the NCBI/GenBank/DDBJ database under accession numbers DRR624878–DRR624895.

## Discussion

This study is the first to identify the bacteria present in centipede forcipules to elucidate the mechanism underlying NSTIs caused by centipede bites. Although NSTIs are rare, they are critical diseases, and the possibility of bacterial transition from centipede forcipules cannot be ignored. Our findings demonstrate for the first time that centipede forcipules harbor many bacteria.

NSTIs occurring after centipede bites have been reported, and arthropod bites are considered a risk factor for NSTI [19]. However, the causes of these infections remain unknown. Several possibilities were considered. First, bacteria in the venom glands of centipede forcipules enter the human body at the time of bite. Second, when observed under a microscope, the surfaces of the centipede forcipules exhibit minute irregularities, and bacteria attached to these forcipules may enter the body when the centipede bites. Third, bacteria that normally reside on the human skin and are unable to penetrate the skin barrier can be introduced into the body through a centipede’s bite and then multiply and spread at the site of the bite. Finally, more potent bacteria from external sources may invade areas with exudate leakage or mild bacterial infection, multiply, and colonize the wound.

Centipede forcipules house venom glands. Accordingly, using forcipules as specimens, we surveyed bacteria not only on the forcipular surface but also within the venom glands. We considered that bacteria present on the forcipules and in the glands may enter the human body with a bite and cause infection, reflecting the first and second possibilities.

This study is not the first to propose that the bacteria present in biting teeth, spines, and venom glands cause infection. Soft tissue infections caused by resident bacteria in the oral cavity of cats and dogs have been reported after bite accidents involving these animals [20,21]. The surface of the fangs and salivary glands of *Varanus komodoensis* may contain bacterial flora that cause sepsis [22]. Additionally, sand flies egest intestinal bacteria (microorganisms) when they feed (egested into the feeding substrates) [23]. After studying the envenomation organs and venom microbiota of snake and spider species [24], it would not be surprising to find that centipedes also have bacteria that can cause infections in their venom claws, glands, and intestines. Antibacterial peptides existing on the body surface of centipedes [8–10] may lead to bacterial selection.

When the venom glands and claws were analyzed at the same time as the centipede forcipules by 16S rRNA sequence analysis following incubation on a blood agar plate, bacteria reported as major monomicrobial causes of NSTI (*Streptococcus pyogenes, Staphylococcus aureus,* and *Escherichia coli*) [18] were not detected, although *Staphylococcus* sp. and *Pseudomonas* sp. were detected in some samples. Using comprehensive 16S rRNA gene sequencing, we isolated a few of the genus_staphylococcus as those found in past infections following centipede bites [5,6]. Additionally, because intestinal contents may be expelled during feeding, we examined the intestinal contents of the centipedes. The bacteria cultured from intestinal contents differed from those isolated from infections after a centipede bite. In previously published studies on the gut microbiota of centipedes [25], the bacteria isolated from centipede bite wounds were not identified as part of the dominant microbiota. In contrast, the causative bacteria detected in severe soft tissue infections after centipede bites were commensal bacteria of the human skin [4–7]. Infections after centipede bites are likely caused by the introduction of skin flora into the body by centipede forcipules during the bite or by some bacterial types such as methicillin-resistant *Staphylococcus aureus*, which become dominant in the wound after the centipede bite. Since the bacteria reported in previous cases of severe soft tissue infections following centipede bites were scarcely detected in this study, it is unlikely that it was resident bacteria in centipede forcipules, including venom glands, that invaded the human body and caused the infection. However, in cases of severe soft tissue infections that occur after centipede bites, wound culture should be performed. We did not completely rule out the possibility that bacteria present on forcipules do not cause soft tissue infection.

Herein, we observed a discrepancy in the bacterial communities detected by culture on blood agar and by microbiome analysis based on 16S rRNA gene sequencing. Similar mismatches have been reported previously, but their underlying causes have not yet been fully understood [26,27]. This discrepancy may, at least in part, be attributable to the fundamentally different aims and selection biases of molecular and culture-based approaches: comprehensive 16S rRNA gene sequencing-based microbiome profiling seeks to capture the *in situ* microbial community as comprehensively as possible, whereas blood-agar culture selectively amplifies only those organisms that grow well under the specific medium and incubation conditions, making a direct comparison between the two inherently difficult. In fact, some of the bacterial taxa detected only by comprehensive 16S rRNA gene sequencing in our study have been reported to have optimal growth temperatures below 30°C [28–30]. Furthermore, members of the family *Acetobacteraceae* have been described as nutritionally demanding and difficult to isolate and cultivate on artificial media [29]. The genus *Methylobacterium* has also been reported to be slow-growing; it is therefore possible that the incubation period on blood agar in our study was too short to allow visible colony formation [30]. Taken together, such differences in culture conditions required by different bacteria may represent one of the reasons for the divergence in the results of incubation on blood agar plates and those of the analysis of the bacterial community using comprehensive 16S rRNA gene sequencing in the present study. Other potential issues include bias introduced by primers and the possibility that dominant bacterial DNA in samples with low DNA content may be preferentially amplified. To address these potential issues, various approaches have been attempted, such as the use of multiple broad-range primers [31]. Improvements in these methods help resolve this issue.

No special prophylactic antibiotics are required after centipede bites [32]; however, keeping the wound clean by washing it may be important, as various bacteria reside on centipede forcipules. Additionally, in cases of poor response to treatment or unexplained complications during treatment for soft tissue infection after a centipede bite, the potential involvement of bacteria present in the centipede forcipules may be worth considering. In clinical practice, it would be better to collect information on the location where the patient sustained the centipede bite. However, this study did not examine centipedes outside Japan. Previously reported cases of necrotizing skin and soft tissue infection following centipede bites originated from France, Turkey, Italy, and Japan [4–7]. Therefore, trends in Asian countries other than Japan and Europe are issues that require future consideration.

This study had some limitations. First, the centipedes were not processed immediately after capture; some were kept in the laboratory for several days before their forcipules were analyzed. Changes in bacterial flora may have occurred during this period. Second, the study included a small population (14 centipedes from three species). Increasing this small sample size could lead to different findings. However, capturing centipedes in the wild is challenging and poses a practical constraint.

## Conclusion

We examined the commensal bacteria on centipede forcipules and venom glands. We found that the bacteria commonly associated with NSTIs are unlikely to be selectively present. However, if an infection persists despite treatment, reconsidering the antimicrobial regimen may be necessary, considering the diversity of the commensal bacteria on centipede forcipules.

## References

[1] Bonato L, Chagas JA, Edgecombe GD. Chilobase 2.0 - A World Catalogue of Centipedes (Chilopoda). 2016. http://chilobase.biologia.unipd.it

[2] Otten EJ. Venomous animal injuries. In: Marx JA, Rosen P. Rosen’s emergency medicine: concepts and clinical practice. 8th ed. Philadelphia: Elsevier/Saunders. 2014. 239–46.

[3] Niruntarai S, Rueanpingwang K, Othong R. Patients with centipede bites presenting to a university hospital in Bangkok: a 10-year retrospective study. Clin Toxicol (Phila). 2021;59(8):721–6. doi: 10.1080/15563650.2020.1865543 33475426

[4] Serinken M, Erdur B, Sener S, Kabay B, Cevik A. A case of mortal necrotizing fasciitis of the trunk resulting from a centipede (Scolopendra moritans) bite. Internet J Emerg Med. 2005;2:1–5.

[5] Puzzo A, Pari C, Bettinelli G, Raggini F, Paderni S, Belluati A. An unusual two-stage infection following a scolopendra bite. Acta Biomed. 2020;91(14-S):e2020009. doi: 10.23750/abm.v91i14-S.10783 33559643 PMC7944685

[6] Uzel A-P, Steinmann G, Bertino R, Korsaga A. Necrotizing fasciitis and cellulitis of the upper limb resulting from centipede bite: two case reports. Chir Main. 2009;28(5):322–5. doi: 10.1016/j.main.2009.05.001 19574077

[7] Tanaka Y, Mato T, Fujiya S, Furuhashi Y, Takanosu T, Watanabe N, et al. Necrotizing Soft-Tissue Infection of the Trunk Resulting From Wound Caused by a Centipede: A Case Report. Am J Case Rep. 2022;23:e937869. doi: 10.12659/AJCR.937869 36350797 PMC9662074

[8] Chaparro E, da Silva PI Jr. Lacrain: the first antimicrobial peptide from the body extract of the Brazilian centipede Scolopendra viridicornis. Int J Antimicrob Agents. 2016;48(3):277–85. doi: 10.1016/j.ijantimicag.2016.05.015 27451089

[9] Wenhua R, Shuangquan Z, Daxiang S, Kaiya Z, Guang Y. Induction, purification and characterization of an antibacterial peptide scolopendrin I from the venom of centipede Scolopendra subspinipes mutilans. Indian J Biochem Biophys. 2006;43(2):88–93. 16955756

[10] Peng K, Kong Y, Zhai L, Wu X, Jia P, Liu J, et al. Two novel antimicrobial peptides from centipede venoms. Toxicon. 2010;55(2–3):274–9. doi: 10.1016/j.toxicon.2009.07.040 19716842

[11] Illumina. MiSeq Sequencing Protocol. https://support.illumina.com/documents/documentation/chemistry_documentation/16s/16s-metagenomic-library-prep-guide-15044223-b.pdf

[12] Bolyen E, Rideout JR, Dillon MR, Bokulich NA, Abnet CC, Al-Ghalith GA, et al. Reproducible, interactive, scalable and extensible microbiome data science using QIIME 2. Nat Biotechnol. 2019;37(8):852–7. doi: 10.1038/s41587-019-0209-9 31341288 PMC7015180

[13] Callahan BJ, McMurdie PJ, Rosen MJ, Han AW, Johnson AJA, Holmes SP. DADA2: High-resolution sample inference from Illumina amplicon data. Nat Methods. 2016;13(7):581–3. doi: 10.1038/nmeth.3869 27214047 PMC4927377

[14] Quast C, Pruesse E, Yilmaz P, Gerken J, Schweer T, Yarza P, et al. The SILVA ribosomal RNA gene database project: improved data processing and web-based tools. Nucleic Acids Res. 2013;41(Database issue):D590-6. doi: 10.1093/nar/gks1219 23193283 PMC3531112

[15] Jordan EB. qiime2R: importing QIIME2 artifacts and associated data into R sessions. GitHub. 2018. https://github.com/jbisanz/qiime2R

[16] McMurdie P, Phyloseq HS. An R package for reproducible interactive analysis and graphics of microbiome census data. 2013. 10.1371/journal.pone.0061217 PMC3632530 23630581

[17] Xu S, Zhan L, Tang W, Wang Q, Dai Z, Zhou L, et al. MicrobiotaProcess: A comprehensive R package for deep mining microbiome. Innovation (Camb). 2023;4(2):100388. doi: 10.1016/j.xinn.2023.100388 36895758 PMC9988672

[18] Kulasegaran S, Cribb B, Vandal AC, McBride S, Holland D, MacCormick AD. Necrotizing fasciitis: 11-year retrospective case review in South Auckland. ANZ J Surg. 2016;86(10):826–30. doi: 10.1111/ans.13232 26211758

[19] Stevens DL, Bryant AE. Necrotizing Soft-Tissue Infections. N Engl J Med. 2017;377(23):2253–65. doi: 10.1056/NEJMra1600673 29211672

[20] Talan DA, Citron DM, Abrahamian FM, Moran GJ, Goldstein EJ. Bacteriologic analysis of infected dog and cat bites. Emergency Medicine Animal Bite Infection Study Group. N Engl J Med. 1999;340(2):85–92. doi: 10.1056/NEJM199901143400202 9887159

[21] Bertin N, Brosolo G, Pistola F, Pelizzo F, Marini C, Pertoldi F, et al. *Capnocytophaga canimorsus*: an emerging pathogen in immunocompetent patients-experience from an emergency department. J Emerg Med. 2018;54: 871–5. 29523423 10.1016/j.jemermed.2018.01.042

[22] Montgomery JM, Gillespie D, Sastrawan P, Fredeking TM, Stewart GL. Aerobic salivary bacteria in wild and captive Komodo dragons. J Wildl Dis. 2002;38(3):545–51. doi: 10.7589/0090-3558-38.3.545 12238371

[23] Dey R, Joshi AB, Oliveira F, Pereira L, Guimarães-Costa AB, Serafim TD, et al. Gut Microbes Egested during Bites of Infected Sand Flies Augment Severity of Leishmaniasis via Inflammasome-Derived IL-1β. Cell Host Microbe. 2018;23(1):134-143.e6. doi: 10.1016/j.chom.2017.12.002 29290574 PMC5832060

[24] Esmaeilishirazifard E, Usher L, Trim C, Denise H, Sangal V, Tyson GH, et al. Bacterial Adaptation to Venom in Snakes and Arachnida. Microbiol Spectr. 2022;10(3):e0240821. doi: 10.1128/spectrum.02408-21 35604233 PMC9248900

[25] Vahtera V, Rezola U, Duplouy A. Bacterial diversity associated with the brown stone centipede, Lithobius forficatus (Chilopoda, Lithobiomorpha). Ann Zool Fenn. 2024;61:33–45.

[26] Pearce MM, Hilt EE, Rosenfeld AB, Zilliox MJ, Thomas-White K, Fok C, et al. The female urinary microbiome: a comparison of women with and without urgency urinary incontinence. mBio. 2014;5(4):e01283-14. doi: 10.1128/mBio.01283-14 25006228 PMC4161260

[27] Hammoudeh Y, Suresh L, Ong ZZ, Lister MM, Mohammed I, Thomas DJI, et al. Microbiological culture versus 16S/18S rRNA gene PCR-sanger sequencing for infectious keratitis: a three-arm, diagnostic cross-sectional study. Front Med (Lausanne). 2024;11:1393832. doi: 10.3389/fmed.2024.1393832 39206175 PMC11352289

[28] Petrus AK, Rutner C, Liu S, Wang Y, Wiatrowski HA. Mercury Reduction and Methyl Mercury Degradation by the Soil Bacterium Xanthobacter autotrophicus Py2. Appl Environ Microbiol. 2015;81(22):7833–8. doi: 10.1128/AEM.01982-15 26341208 PMC4616949

[29] Gomes RJ, Borges MF, Rosa MF, Castro-Gómez RJH, Spinosa WA. Acetic Acid Bacteria in the Food Industry: Systematics, Characteristics and Applications. Food Technol Biotechnol. 2018;56(2):139–51. doi: 10.17113/ftb.56.02.18.5593 30228790 PMC6117990

[30] Kovaleva J, Degener JE, van der Mei HC. Methylobacterium and its role in health care-associated infection. J Clin Microbiol. 2014;52(5):1317–21. doi: 10.1128/JCM.03561-13 24430456 PMC3993692

[31] Schulze-Schweifing K, Banerjee A, Wade WG. Comparison of bacterial culture and 16S rRNA community profiling by clonal analysis and pyrosequencing for the characterization of the dentine caries-associated microbiome. Front Cell Infect Microbiol. 2014;4:164. doi: 10.3389/fcimb.2014.00164 25429361 PMC4228914

[32] Changratanakorn C, Fasawang N, Chenthanakit B, Tansanthong P, Mapairoje C, Tunud R, et al. Effectiveness of antibiotic prophylaxis in patients with centipede stings: a randomized controlled trial. Clin Exp Emerg Med. 2021;8(1):43–7. doi: 10.15441/ceem.20.110 33845522 PMC8041584

